# Body mass index modifies the relationship between γ-H2AX, a DNA damage biomarker, and pathological complete response in triple-negative breast cancer

**DOI:** 10.1186/s12885-016-3045-z

**Published:** 2017-02-06

**Authors:** Maddalena Barba, Patrizia Vici, Laura Pizzuti, Luigi Di Lauro, Domenico Sergi, Anna Di Benedetto, Cristiana Ercolani, Francesca Sperati, Irene Terrenato, Claudio Botti, Lucia Mentuccia, Laura Iezzi, Teresa Gamucci, Clara Natoli, Ilio Vitale, Marcella Mottolese, Ruggero De Maria, Marcello Maugeri-Saccà

**Affiliations:** 1 Division of Medical Oncology 2, “Regina Elena” National Cancer Institute, Via Elio Chianesi 53, 00144 Rome, Italy; 2 Scientific Direction, “Regina Elena” National Cancer Institute, Rome, Italy; 3 Department of Pathology, “Regina Elena” National Cancer Institute, Rome, Italy; 4 Biostatistics-Scientific Direction, “Regina Elena” National Cancer Institute, Rome, Italy; 5 Department of Surgery, “Regina Elena” National Cancer Institute, Rome, Italy; 6 Medical Oncology Unit, ASL Frosinone, Frosinone, Italy; 7 Department of Medical, Oral and Biotechnological Sciences, University “G. d’Annunzio”, Chieti, Italy; 8 Department of Biology, University of Rome “Tor Vergata”, Rome, Italy; 9 Institute of General Pathology, Catholic University of the Sacred Heart, Largo Agostino Gemelli, 10, 00168 Rome, Italy

**Keywords:** Body mass index, γ-H2AX, Chk1, Double-strand breaks, Pathological complete response, Triple-negative breast cancer

## Abstract

**Background:**

Body mass index (BMI) is largely investigated as a prognostic and predictive factor in triple-negative breast cancer (TNBC). Overweight and obesity are linked to a variety of pathways regulating tumor-promoting functions, including the DNA damage response (DDR). The DDR physiologically safeguards genome integrity but, in a neoplastic background, it is aberrantly engaged and protects cancer cells from chemotherapy. We herein verified the role of BMI on a previously assessed association between DDR biomarkers and pathological complete response (pCR) in TNBC patients treated with neoadjuvant chemotherapy (NACT).

**Methods:**

In this retrospective analysis 54 TNBC patients treated with NACT were included. The relationship between DDR biomarkers, namely phosphorylated H2A Histone Family Member X (γ-H2AX) and phosphorylated checkpoint kinase 1 (pChk1), and pCR was reconsidered in light of BMI data. The Pearson’s Chi-squared test of independence (2-tailed) and the Fisher Exact test were employed to assess the relationship between clinical-molecular variables and pCR. Uni- and multivariate logistic regression models were used to identify variables impacting pCR. Internal validation was carried out.

**Results:**

We observed a significant association between elevated levels of the two DDR biomarkers and pCR in patients with BMI < 25 (*p* = 0.009 and *p* = 0.022 for γ-H2AX and pChk1, respectively), but not in their heavier counterpart. Results regarding γ-H2AX were confirmed in uni- and multivariate models and, again, for leaner patients only (γ-H2AX^high^ vs γ-H2AX^low^: OR 10.83, 95% CI: 1.79–65.55, *p* = 0.009). The consistency of this finding was confirmed upon internal validation.

**Conclusions:**

The predictive significance of γ-H2AX varies according to BMI status. Indeed, elevated levels of γ-H2AX seemed associated with lower pCR rate only in leaner patients, whereas differences in pCR rate according to γ-H2AX levels were not appreciable in heavier patients. Larger investigations are warranted concerning the potential role of BMI as effect modifier of the relationship between DDR-related biomarkers and clinical outcomes in TNBC.

## Background

Overwhelming evidence connects obesity with breast cancer (BC) [1, 2]. In particular, obesity is increasingly designated as a risk factor for triple-negative BC (TNBC) [3–8].

Preclinical models have provided ground for the role of cellular metabolism and energy balance in affecting cancer progression and, ultimately, therapeutic outcomes [9]. The hormonal milieu underling obesity is complex. In obese patients, the altered dynamics of insulin secretion translates into increased levels of insulin and insulin-like growth factors. In addition, abnormalities have been described in the expression profiles of various adipokines and cytokines [9]. This abnormal status leads to the activation of oncogenic intracellular molecular networks in cancer cells, such as the JAK2/STAT3, MAPK/ERK, PI3K/AKT and NF-kB pathways [9]. Moreover, the low chronic tissue inflammation status that accompanies obesity enhances the activity of some factors, such as hypoxia-inducible factor 1α (HIF1α), which in turn promotes angiogenesis and acquisition of cancer stem-like traits [10–12].

Next, obesity-related oxidative stress generates reactive oxygen species (ROS), which may outcompete the antioxidant defense systems, thus altering the structure of the DNA and ultimately leading to damages and mutations [13]. In order to deal with endogenous and exogenous sources of DNA damage, preventing the onset and accumulation of sub-lethal genetic lesions, and avoiding lesion amplification upon cellular division, eukaryotic cells are equipped with a tightly regulated machinery, the DNA damage response (DDR) pathway [14]. Through the coordinated recruitment of cell cycle checkpoints, DNA repair mechanisms and apoptotic pathways, the DDR orchestrates repair of DNA lesions, or promote self-elimination of cells whose damages overwhelm repair capacity [14].

In a neoplastic background, the DDR apparatus is aberrantly regulated. Oncogene-induced replication stress and altered cell cycle progression, arising from mutational events in proliferative and cell-cycle control genes, respectively, require an adaptive response to ensure cell viability [15]. In this frame, activation of the Ataxia-Telangiectasia Mutated (ATM)-Checkpoint Kinase 2 (Chk2) and ataxia telangiectasia and Rad3-related protein (ATR)-Checkpoint kinase 1 (Chk1) pathways becomes central [16]. One of the most dramatic implication of the increased ability of cancer cells to correct genetic lesions when exposed to DNA-damaging agents refers to resistance to chemotherapy [17]. Consistently, DNA damage-related biomarkers are the focus of intense investigations for the development of predictive tools, and great expectations are placed on novel drugs able to interfere with DNA repair ability [15].

We have recently reported on the association between elevated levels of phosphorylated H2A Histone Family Member X (γ-H2AX), a marker of DNA double-strand breaks that activate the ATM-Chk2 pathway, and reduced pathological complete response (pCR) rate in TNBC patients treated with neoadjuvant chemotherapy (NACT) [18]. In this cohort, we did not observe a significant association between phosphorylated Chk1 levels and the explored outcome [18].

**Fig. 1:**
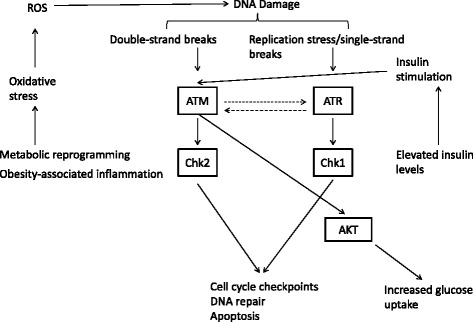
Schematic representation of the relationship between obesity-related alterations and the DDR machinery. The increased production of reactive oxygen species (ROS), stemming from both metabolic reprogramming of cancer cells and the obesity-related inflammatory status (*left*), results in elevated levels of DNA damage (oxidative stress-related DNA damage) with the consequent activation of the ATM and ATR pathways. Moreover, insulin, whose levels increase in obese patients (insulin resistance), activates ATM that in turn increases glucose uptake via AKT (*right*)

Given the connection between obesity and TNBC, and the link between oxidative stress and the DDR at the molecular level (Fig. 1), we herein investigated the impact of body mass index (BMI), a widely used indicator of generalized obesity, on the association between DDR biomarkers and pCR.

## Methods

From the original series of 66 TNBC patients treated with NACT analyzed for studying the predictive significance of γ-H2AX and pChk1 [18], we were able to retrieve BMI data for 54 patients. For this retrospective analysis, patients were considered eligible if all the relevant clinical-molecular information were available, and if the presurgical treatment was completed. Regarding estrogen receptor (ER) and progesterone receptor (PgR), six tumors displayed a weak (≤10%) expression of either ER or PgR in diagnostic biopsies, which became negative (0%) in surgical samples after treatment. These patients were included on the basis of the clinical plausibility of a basal-like portrait of their tumors [19]. BMI was defined using the cutoff suggested by the world health organization (WHO) to distinguish between normal weight (BMI <25) and overweight (BMI ≥25) subjects. NACT consisted in concomitant or sequential anthracycline-taxane-based regimens, as detailed elsewhere [18]. pCR was defined as no residual invasive tumor in both breast and axilla, irrespective of the presence of ductal carcinoma in situ (ypT0/is ypN0). The immunohistochemical assessment of γ-H2AX and pChk1 was performed in formalin-fixed paraffin-embedded (FPPE) tissues with the anti-phospho-H2AX (Ser139) (clone JBW301) mouse monoclonal antibody (MAb) (Upstate) and the anti-phospho-Chk1 (Ser345) (clone 133D3) rabbit MAb (Cell Signaling) [18]. The expression levels of γ-H2AX were evaluated in terms of nuclear-expressing tumor cells and analyzed as a categorical variable. To this end, the median score of all tumors was used to classify low and high expressing samples (γ-H2AX ^low^ and γ-H2AX ^high^) [18]. pChk1 was considered as positive or negative on the basis of nuclear staining intensity (0: negative, 1+: weak, 2+: moderate, 3+: strong). Tumors with absent (0) nuclear staining were considered as negative (pChk1^neg^), and tumors with weak to strong (1–3) nuclear staining were considered as positive (pChk1^pos^) [18]. Immunoreactivity was assessed by two independent investigators (ADB and CE) and discordant cases were reviewed by a third expert (MM). This retrospective study was conducted in accordance with the Declaration of Helsinki and was approved by the Ethic Committee of “Regina Elena” National Cancer Institute, the coordinating centre. Written informed consents were secured before chemotherapy.

### Statistical analysis

Descriptive statistics were computed for all the variables of interest including clinical, pathological, molecular and anthropometric features. To assess the relationship between categorical variables we used the Pearson’s Chi-squared test of independence (2-tailed) and the Fisher Exact test, depending upon the size of groups compared. BMI was computed as weight in kilograms divided by the square of height in meters (kg/m2), and considered as a categorical variable on the basis of the cutoff proposed by the WHO to define normal weight (BMI < 25) and overweight (≥25) patients. Univariate logistic regression model was used to identify variables impacting pCR. A multivariate logistic regression model was built using a stepwise regression approach (forward selection) and the related estimates reported as Odds Ratio (OR) and 95% Confident Interval (CI). The enter and remove limits were *p* = 0.10 and *p* = 0.15, respectively. A multivariate logistic regression model was also generated by including all the variables significant at the univariate assessment. To estimate the risk of an overfitted model, internal validation was performed using a re-sampling without replacement procedure [20, 21]. One hundred datasets were generated by randomly removing approximately 20% of the original sample and the replication rate was calculated. We considered statistically significant *p* values less than 0.05. Statistical analyses were carried out using SPSS software (SPSS version 21, SPSS Inc., Chicago, IL, USA).

## Results

Cancer- and patient-related features are summarized in Table 1. In this series of 54 TNBC patients, 31 (57.4%) patients had a BMI < 25. With the exception of an association between BMI < 25 and younger age at diagnosis, we did not observe any further relationship between BMI and clinical-molecular features, DDR biomarkers and pCR (Table 2). Likewise, neither γ-H2AX nor pChk1 were associated with clinical-molecular features (data available upon request).

**Table 1:** Baseline characteristics and treatment outcome of TNBC patients treated with neoadjuvant chemotherapy (*N* = 54)

Age at diagnosis	
median (min-max) [IQrange]	49.2 (26.7–76.6) [45.3–60.3]
<49	25 (46.3)
≥49	29 (53.7)
Stage	
II	18 (33.3)
III	36 (66.7)
Grade	
1–2	22 (40.7)
3	32 (59.3)
Ki-67	
median (min-max) [IQrange]	70.0 (10.0–90.0) [43.7–80.0]
Chemotherapy	
Sequential	47 (87.0)
Concomitant	7 (13.0)
pCR	
No	37 (68.5)
Yes	17 (31.5)
BMI	
median (min-max) [IQrange]	23.9 (17.5–41.6) [21.7–25.9]
< 25	31 (57.4)
≥ 25	23 (42.6)
γ-H2AX	
Low	25 (46.3)
High	29 (53.7)
pChk1	
Neg	16 (29.6)
Pos	38 (70.4)

**Table 2:** Association between BMI and clinical-molecular features (*N* = 54)

	BMI		Chi2 Test
	<25	≥25	*p*-value
	N (%)	N (%)	
Age at diagnosis			
< 49	20 (80.0)	5 (20.0)	0.002
≥ 49	11 (37.9)	18 (62.1)	
Stage			
II	12 (66.7)	6 (33.3)	0.331
III	19 (52.8)	17 (47.2)	
Grade			
1–2	13 (59.1)	9 (40.9)	0.836
3	18 (56.3)	14 (43.8)	
Ki-67			
Low	15 (60.0)	10 (40.0)	0.721
High	16 (55.2)	13 (44.8)	
Chemotherapy			
Sequential	27 (57.4)	20 (42.6)	0.999^a^
Concomitant	4 (57.1)	3 (42.9)	
pCR			
No	19 (51.4)	18 (48.6)	0.184
Yes	12 (70.6)	5 (29.4)	
γ-H2AX			
Low	16 (64.0)	9 (36.0)	0.363
High	15 (51.7)	14 (48.3)	
pChk1			
Neg	12 (75.0)	4 (25.0)	0.090
Pos	19 (50.0)	19 (50.0)	

^a^Fisher’s Exact Test

Although the sample size was slightly smaller compared with the original cohort [18], consistently with our previous results, elevated γ-H2AX levels retained significant association with reduced pCR rate (*p* = 0.015), and a suggestion towards an association between pChk1 and pCR was also observed (*p* = 0.057) (data available upon request).

When stratifying by BMI, the association between DNA damage biomarkers and pCR was not appreciable in patients with BMI ≥ 25 (Table 3). Conversely, in leaner patients, namely patients with a BMI < 25, elevated levels of γ-H2AX and pChk1 predicted lower pCR rate (Table 3). Uni- and multivariate analyses confirmed the predictive ability of γ-H2AX in leaner patients (γ-H2AX^high^ vs γ-H2AX^low^: OR 10.83, 95% CI: 1.79–65.55, *p* = 0.009), but not in patients with BMI ≥25 (Table 4). The replication rate of the model in leaner patients was 87%. This data indicates that the association between higher levels of γ-H2AX and lower pCR rate tested significant in 87 out of 100 replications. In the multivariate model adjusted by variables testing significant at univariate assessment, the association between γ-H2AX and pCR was borderline significant in patients with BMI < 25 (Table 5).

**Table 3:** Association between DDR biomarkers and pCR in TNBC patients with BMI < 25 and BMI ≥ 25 (*N* = 54)

	BMI < 25				BMI ≥ 25	
	No pCR	pCR	Fisher’s Exact Test	No pCR	pCR	Fisher’s Exact Test
	N (%)	N (%)	*p*-value	N (%)	N (%)	*p*-value
pCHK1						
Neg	4 (33.3)	8 (66.7)	0.022	4 (100.0)	0 (0.0)	0.539
Pos	15 (78.9)	4 (21.1)		14 (73.7)	5 (26.3)	
γ-H2AX						
low	6 (37.5)	10 (62.5)	0.009	7 (77.8)	2 (22.2)	0.999
high	13 (86.7)	2 (13.3)		11 (78.6)	3 (21.4)

**Table 4:** Uni- and multivariate logistic regression models of patient- and disease-related features and pathological complete response (*N* = 54)

	BMI < 25					
	Univariate logistic regression			Multivariate logistic regression^a^		
	OR	95%CI	*p*-value	OR	95%CI	*p*-value
Stage						
III vs II	0.37	0.08–1.81	0.220			
Grade						
3 vs 1–2	0.98	0.23–4.25	0.981			
Ki-67						
High vs Low	0.19	0.04–0.97	0.046			
γ-H2AX						
High vs Low	10.83	1.79–65.55	0.009	10.83	1.79–65.55	0.009
pChk1						
Pos vs Neg	7.50	1.47–38.28	0.015			
	BMI ≥ 25					
	Univariate logistic regression			Multivariate logistic regression		
	OR	95%CI	*p*-value	OR	95%CI	*p*-value
Stage						
III vs II	0.65	0.06–7.32	0.727			
Grade						
3 vs 1–2	3.00	0.39–23.07	0.291			
Ki-67						
High vs Low	0.25	0.02–2.70	0.253			
γ-H2AX						
High vs Low	1.05	0.14–7.93	0.964			
pChk1						
Pos vs Neg	Not applicable					

^a^with forward stepwise inclusion

**Table 5:** Uni- and multivariate logistic regression models of patient- and disease-related features and pCR upon adjustment of the multivariate model for Ki-67, γ-H2AX and pChk1 (*N* = 54)

	BMI < 25					
	Univariate logistic regression			Multivariate logistic regression^a^		
	OR	95%CI	*p*-value	OR	95%CI	*p*-value
Stage						
III vs II	0.37	0.08–1.81	0.220			
Grade						
3 vs 1–2	0.98	0.23–4.25	0.981			
Ki67						
High vs Low	0.19	0.04–0.97	0.046	0.30	0.04–2.06	0.223
γ-H2AX						
High vs Low	10.83	1.79–65.55	0.009	6.34	0.89–45.33	0.066
pChk1						
Pos vs Neg	7.50	1.47–38.28	0.015	4.82	0.77–30.26	0.093
	BMI ≥ 25					
	Univariate logistic regression			Multivariate logistic regression		
	OR	95%CI	*p*-value	OR	95%CI	*p*-value
Stage						
III vs II	0.65	0.06–7.32	0.727			
Grade						
3 vs 1–2	3.00	0.39–23.07	0.291			
Ki-67						
High vs Low	0.25	0.02–2.70	0.253			
γ-H2AX						
High vs Low	1.05	0.14–7.93	0.964			
pChk1						
Pos vs Neg	Not applicable					

^a^Adjusted for: Ki-67, γ-H2AX and pChk1

## Discussion

The aim of the present study was to assess the role of BMI on the previously verified association between DDR biomarkers and pCR rate in a historic cohort of TNBC patients treated with NACT. We observed a significant association between elevated levels of γ-H2AX and reduced pCR rate in leaner patients. A similar suggestion was observed for pChk1, albeit at a not fully significant extent.

The achievement of pCR in TNBC is an extremely relevant clinical goal, considering that this intermediate endpoint is tied to long-term survival outcomes. In this view, the search for biomarkers foreseeing sensitivity/resistance to NACT is of paramount importance [22, 23]. Over time, a variety of potential DDR-related biomarkers have been proposed, with inconsistent results. However, in previous studies the focus was mostly placed on single endpoints acting in the context of distal DDR effectors, such as the excision repair cross-complementation group1 (ERCC1) protein [24, 25]. Coherently with our preclinical findings describing Chk1 as a crucial mediator of chemotherapy resistance in patient-derived CSC models and xenografts [26], we decided to investigate key DDR pathway components deputed to initiate cell cycle arrest upon DNA damage.

The use of a retrospective study design, particularly in a moderately-sized cohort, invites caution in results interpretation. Nevertheless, these findings hold a potential in generating hypotheses on how the host metabolic status may be linked to specific cancer-related functions and therapeutic outcomes. Thus, our results provided ground for preclinical studies addressing the connection between specific metabolic pathways, and obesity-related molecular changes, and the biology of TNBC.

As briefly aforementioned, anthropometric features and particularly BMI, have been the focus of considerable attention in TNBC. Nevertheless, conflicting results were reported when BMI was analyzed as a potential prognostic factor. Tait et al. did not observe any effect of BMI and diabetes on survival outcomes [27], whereas Hao et al. [28] and Cakar et al. [29] observed that overweight is associated with adverse outcomes in TNBC, consistently with the findings reported by Widschwendter in the case of severe obesity (BMI ≥ 40) [30]. Regarding the association between BMI and pCR, a pooled analysis including patients from eight neoadjuvant trials verified the detrimental effect of overweight and obesity on survival outcomes, but not on pCR, in TNBC. However, when considering the overall study population (8872 patients), BMI significantly impacted both pCR and survival [31]. Overall, data on BMI and metabolic determinants as predictive/prognostic factors in TNBC are still in their infancy. To this end, our data add an important piece to the puzzle, suggesting that DNA repair proficiency of TNBC cells may vary in relation to metabolic cues. Our data seem to indicate the existence of an inverse association between elevated levels of γ-H2AX and reduced pCR rate in leaner patients only. Study weaknesses mainly stemming from the quite restricted sample size and study design, i.e., retrospective case series, refrained us from conducting subgroup analysis within each BMI category. In future and adequately sized studies, informative details may come from characterizing the distribution of TNBC molecular subtypes across BMI strata along with a more extensive definition of the metabolic profile of the host. In more details, two strategies should be pursued in our opinion. First, TNBC is a heterogeneous disease [32]. Gene expression profiles revealed the existence of multiple molecular entities [32]. For instance, a luminal androgen receptor (LAR) subtype was identified and characterized for the enrichment of hormonally regulated pathways, such as those involved in steroid synthesis and androgen/estrogen metabolism. Consistently, a great interest surrounds the use of antiandrogens in TNBC expressing the androgen receptor, and preliminary clinical data support the therapeutic relevance of androgen receptor targeting in this disease [33]. Conversely, the basal-like 1 subtype is characterized by the expression of DNA damage response pathways, together with genes associated with proliferation and cell cycle checkpoints [32]. On this basis, we can speculate that the host metabolic status might have a different significance across the constellation of TNBC subtypes, and that metabolic avenues might specifically be linked to some TNBC subtype, without transversally influencing all the disease entities encompassed into the definition of TNBC. If this is the case, the different “metabolic dependency” of various TNBC subtypes may, at least partly, account for the effect of BMI on the predictive ability of DDR biomarkers reported in the present study.

Second, dissecting the link between metabolic abnormalities, specific molecular pathways, and clinical outcomes based on the exclusive consideration of BMI probably might represent an oversimplification. Accordingly, we have implemented our research agenda on metabolic factors in BC [34–37], which now includes a deeper characterization of the metabolic status in patients whose tumors will be evaluated for candidate molecular biomarkers. The molecular analysis of pathways potentially connected with therapeutic resistance will be integrated by an extensive metabolic characterization, which includes: i) prospective collection of anthropometric data using standardized operative procedures (SODs) and inclusion of waist circumference, which is more tightly related to visceral adiposity and more strongly associated with multiple chronic diseases by underlying metabolic alterations [38], ii) dual-energy X-ray absorptiometry (DEXA) to calculate the percent of body fat in the visceral and subcutaneous compartments, iii) homeostatic model assessment (HOMA) index for assessing insulin resistance, and iv) fasting glucose, insulin levels and lipidic profile including total and fractionated cholesterol. The combination of information collected both at the tissue and systemic level will help depict a more comprehensive scenario on the influence of metabolic determinants on TNBC, and will thus possibly represent the starting point for larger, prospective studies.

## Conclusions

The predictive ability of DDR biomarkers in TNBC patients who received NACT seems to be significantly affected by BMI, with the highest predictive performance of the biomarkers of interest being achieved for patients with BMI < 25. Based on the promising nature of these results, future translational studies within this pipeline may be greatly implemented by the prospective and standardized collection of anthropometrics including BMI, a widely accepted indicator of general adiposity, along with waist circumference, which better captures visceral adiposity. Anthropometric data will be efficiently integrated by circulating biomarkers of energy metabolism. In addition, the metabolic study may be further and easily enriched by DEXA scans for body composition. The systematic evaluation of the metabolic asset of the host will be then weighted against the molecular portrait of the specific molecular subtypes of TNBC. As a likely result, the combination of metabolic and molecular pieces will display an entirely renewed puzzle which will help address the clinical significance of deregulated pathway nodes, especially when they are potentially affected by the metabolic milieu of the patients. In conclusion, larger studies, envisioning the molecular characterization of TNBC coupled with an extensive assessment of the host metabolic status, are warranted to provide novel insights into this fascinating topic.

## Abbreviations

ATM: Ataxia-telangiectasia mutated
ATR: Ataxia telangiectasia and Rad3-related protein
BC: Breast cancer
BMI: Body mass index
Chk2: Checkpoint Kinase 2
DDR: DNA damage
ER: Estrogen receptor
NACT: Neoadjuvant chemotherapy
pChk1: Phosphorylated checkpoint kinase 1
pCR: Pathological complete response
PgR: Progesterone receptor
ROS: Reactive oxygen species
TNBC: Triple negative breast cancer
WHO: World health organization
γ-H2AX: Phosphorylated H2A Histone Family Member X
